# Dried Volumetric Microsampling Approaches for the Therapeutic Drug Monitoring of Psychiatric Patients Undergoing Clozapine Treatment

**DOI:** 10.3389/fpsyt.2022.794609

**Published:** 2022-06-01

**Authors:** Camilla Marasca, Roberto Mandrioli, Roccaldo Sardella, Tomaž Vovk, Andrea Armirotti, Andrea Cavalli, Alessandro Serretti, Michele Protti, Laura Mercolini

**Affiliations:** ^1^Research Group of Pharmaco-Toxicological Analysis (PTA Lab), Department of Pharmacy and Biotechnology (FaBiT), Alma Mater Studiorum – University of Bologna, Bologna, Italy; ^2^Computational and Chemical Biology, Fondazione Istituto Italiano di Tecnologia (IIT), Genoa, Italy; ^3^Department for Life Quality Studies (QuVi), Alma Mater Studiorum – University of Bologna, Rimini, Italy; ^4^Department of Pharmaceutical Sciences, University of Perugia, Perugia, Italy; ^5^Faculty of Pharmacy, University of Ljubljana, Ljubljana, Slovenia; ^6^Analytical Chemistry Lab, Fondazione Istituto Italiano di Tecnologia (IIT), Genoa, Italy; ^7^Department of Pharmacy and Biotechnology (FaBiT), Alma Mater Studiorum – University of Bologna, Bologna, Italy; ^8^Department of Biomedical and Neuromotor Sciences (DIBINEM), Alma Mater Studiorum – University of Bologna, Bologna, Italy

**Keywords:** clozapine, microsampling, volumetric absorptive microsampling (VAMS), microfluidic channel-based technology, therapeutic drug monitoring (TDM)

## Abstract

Clozapine is one of the most widely used second-generation antipsychotic drugs (SGAs) for the treatment of schizophrenia. Despite advantages over first-generation drugs, clozapine still shows significant side effects and interindividual variations in efficacy. In order to ensure frequent therapeutic drug monitoring (TDM) and improve the compliance of psychiatric patients undergoing clozapine treatment, two novel dried microsampling approaches based on whole blood and plasma volumetric absorptive microsampling (b-VAMS and p-VAMS) and microfluidic generated-dried blood spot technology (mfDBS) were developed and coupled to HPLC with electrochemical detection (ED). The proposed miniaturized strategies by means of VAMS and microfluidic channel-based devices provide several advantages in terms of collection, storage, and handling compared to classical blood and plasma processing. Satisfactory validation results were obtained for all microsampling platforms, with mean extraction yields >85.1%, precision as relative standard deviation (RSD) < 5.1%, and stability < 4.5% analyte loss after 30 days for p-VAMS; mean extraction yields > 83.4%, precision RSD < 5.4%, and stability < 4.6% analyte loss after 30 days for b-VAMS, and mean extraction yields > 74.0%, precision RSD < 5.6%, and stability < 4.9% analyte loss after 30 days for mfDBS. The original microsampling methodologies have been successfully applied to the blood and plasma collected from five psychiatric patients for the monitoring of the levels of clozapine and its main metabolites, providing robust and reliable quali-quantitative results. Comparisons between results of the two dried microsampling technologies with those obtained by classic fluid plasma analysis were in good agreement and have demonstrated that the proposed miniaturized approaches could be suitable for TDM purposes.

## Introduction

Schizophrenia is a chronic disorder that involves cognitive, mental, and behavioral dysfunctions often divided into positive symptoms such as hallucinations, delusions, disorganized behavior, and agitation, and negative symptoms such as apathy, poverty of speech, emotional withdrawal, anhedonia, unsociability, and difficulty in abstract thinking (1). According to the World Health Organization (WHO), there are currently about 20 million people all over the world affected by schizophrenia; no country or ethnicity appears to be free from this disease. For this reason, schizophrenia represents a serious public health problem (2). Chlorpromazine, a dopamine receptor antagonist, was the first antipsychotic drug introduced into therapy and the first member of the so-called “typical antipsychotic” or “neuroleptic” class. First-generation antipsychotic drugs (FGAs) have been shown to be mostly active against the positive symptoms of schizophrenia. However, their non-selective blocking of different dopamine receptor subtypes can cause several side effects such as parkinsonism and tardive dyskinesia (3, 4); other side effects are attributed to low-affinity interactions with histamine, muscarinic, adrenergic, and serotonergic receptors. Examples of these side effects are: constipation, dry mouth, blurred vision (antimuscarinic), sedation, weight gain (antihistaminic); orthostatic hypotension, tachycardia (anti-adrenergic); weight gain and withdrawal syndrome (serotonergic) (5). More recently, the introduction of “atypical” or second-generation antipsychotic drugs (SGAs) has brought considerable advantages thanks to wider effectiveness spectrum and reduced risk of adverse events. This different therapeutic profile is probably due to SGAs acting with lower/partial affinity on dopaminergic D_2_ receptors and strongly interacting with a variety of serotonin (5-HT) receptors (3, 4).

**FIGURE 1:**
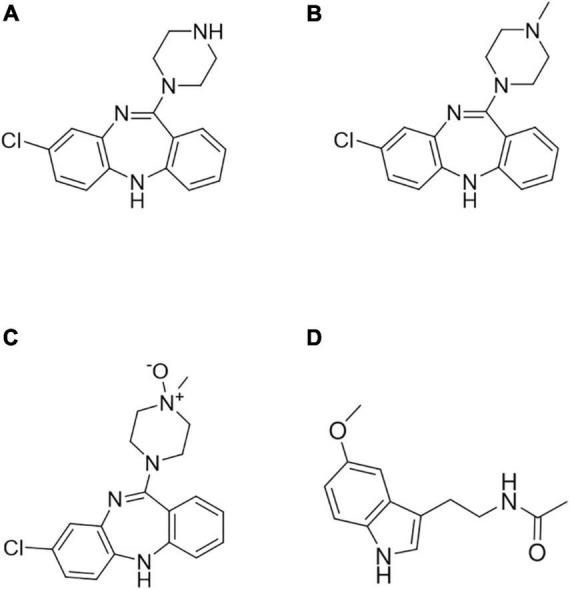
Chemical structures of **(A)** clozapine (CLZ), **(B)** *N*-desmethylclozapine (DMC), **(C)** clozapine *N*-oxide (NOX), and **(D)** melatonin (internal standard, IS).

Clozapine (3-chloro-6-(4-methylpiperazin-1-yl)-11*H*-benzo [b][1,4]benzodiazepine, CLZ, Figure 1A) was the first SGA introduced for the treatment of schizophrenia during the 1970s. Since then, it has demonstrated to be remarkably effective in patients who are “non-responders” to FGAs (6). Although the use of CLZ leads to a lower rate of extrapyramidal side effects and prolactin response, it shows other significant side effects such as agranulocytosis, sedation, orthostatic hypotension, myocarditis, weight gain, and hypersalivation. While plasma CLZ levels in the 250–750 ng/ml range are usually considered therapeutic, the risk of toxic delirium, confusion, and seizures sharply rises at concentrations above 800–1,000 ng/ml (7). Furthermore, pharmacogenetic studies showed high interindividual variations in treatment response and side effects of CLZ associated with metabolic enzyme polymorphisms (8). Additional factors affecting CLZ plasma levels are age, smoking, sex, alcohol and drug abuse, and polypharmacy (9). CLZ is mainly metabolized to *N*-desmethylclozapine (DMC, Figure 1B) by cytochrome P450 (CYP) subtype 1A2 (10) in the liver and to clozapine *N*-oxide (NOX, Figure 1C) by a flavin monooxygenase in the brain. DMC pharmacological activity is shorter and weaker than that of CLZ, while NOX seems to be inactive as an antipsychotic. Both metabolites are found in the plasma at lower levels with respect to CLZ concentration: 50–70% for DMC and 10–20% for NOX (11).

For all these reasons, it is crucial to carry out accurate therapeutic drug monitoring (TDM) of psychiatric patients undergoing CLZ therapy in order to evaluate patient compliance and response, optimize drug dosage, and reduce the risk of adverse effects and dose-related toxicity. According to Consensus Guidelines for Therapeutic Drug Monitoring in Neuropsychopharmacology (12), the level of recommendation to use TDM of clozapine is strongly supported by reports and controlled clinical trials; therefore, the TDM of clozapine is a standard of care. Frequent TDM could help clinical decision-making, aiming at keeping drug levels within the therapeutic window and ensuring treatment efficacy and safety, especially when other central nervous system (CNS) drugs are co-administered (13). Moreover, clinically relevant intra-individual plasma level variability has been observed over time in several patients (14), with at least 50% CV found in 20.7% of patients for CLZ levels and 13.8% of patients for DMC levels in a time span of 1 year. Some authors (15) also suggest that these variations can be linked to lifestyle or physiopathological variables such as tobacco smoking, and body weight.

In order to meet patient and clinician needs, microsampling approaches for TDM are being increasingly exploited, thanks to the possibility of collecting small volumes of capillary whole blood in a minimally invasive way by means of fingerpricking, thus avoiding classic venous blood drawing by phlebotomy. The advantages of miniaturized sampling also concern sample handling and analysis: microsamples allow drying and storage at room temperature (RT) while reducing biohazard risks, improving compound stability profiles thanks to water loss, and facilitating shipping and transport. Moreover, miniaturized samples can allow relatively simple implementation into analytical workflows to develop high-throughput automated procedures (16, 17). The most widespread microsampling approach is represented by dried blood spots (DBSs), which were initially devised for newborn screening of metabolic disorders: some drops of capillary whole blood are deposited onto filter paper cards. Unfortunately, this strategy does not always guarantee sampling accuracy because blood hematocrit (HCT) affects spot size and homogeneity, as well as analytical accuracy (18). To overcome the HCT-dependence of classic DBS, several technologies for volumetric blood microsampling have been proposed (19, 20). In this research, an innovative microfluidic channel-based technology is proposed for consecutive generation of four capillary volumetric blood spots, avoiding potential HCT issues. An accurate and precise volume of 5 or 10 μl can be sampled from a fingertip prick by making the surface of a blood drop come into contact with the microchannel inlet until it is completely filled. Closing the device, an HCT-independent spot is formed on specific DBS cards integrated in the device that can be stored at RT after spot drying. A recent and promising development in DBS is represented by volumetric absorptive microsampling (VAMS), an innovative device consisting of a porous polymeric tip supported by a plastic handle. The tip can absorb an accurate fluid sample volume (10, 20, or 30 μl depending on the format) independently of its density (21). VAMS microsampling maintains all the advantages described above, and it can be exploited for sampling of different fluid matrices and can be implemented in fully automated workflows for complete miniaturization of the entire analytical process with broad applications to bioanalytical fields (22–24). The VAMS technology has been studied for the TDM of different classes of drugs such as antidepressants, antiepileptics, antibiotics, and analgesics, exploiting different biological matrices (25–28). However, microfluidic-generated DBS (mfDBS) technologies have not been widely investigated for TDM purposes (29–31).

To the best of our knowledge, although microsampling techniques have indeed been applied to CLZ TDM before (32–38), VAMS-based microsampling approaches and mfDBS technologies have not yet been investigated; the only example of a microfluidic device used for this purpose is a very recently described sensing platform which, however, is not used to quantify CLZ metabolites (39). In this study, novel analytical methodologies involving innovative blood and plasma VAMS (b-VAMS and p-VAMS) and mfDBS strategies coupled to an effective HPLC method coupled to electrochemical detection (ED, amperometric) were developed, validated, and applied for the analysis of CLZ and its main metabolites in dried blood and plasma microsamples for TDM purposes.

## Experiment

### Chemicals and Chromatographic Conditions

CLZ, DMC, NOX, and melatonin used as internal standard (IS, Figure 1D), all pure powders (>99% purity) were purchased from Sigma-Aldrich (Milan, Italy). HPLC-grade (>99.8%) methanol (MeOH) and acetonitrile (ACN), monobasic sodium phosphate (KH_2_PO_4_), potassium chloride (KCl), 85% (w/w) phosphoric acid, and triethylamine (99%), all pure for analysis, were bought from Sigma-Aldrich (Milan, Italy). Ultrapure water (18.2 MΩ cm) was obtained in-house by means of a MilliQ apparatus by Millipore (Milford, MA, United States). Stock solutions of analytes and IS (1 mg/ml) were prepared in MeOH and stored at −20°C. Fresh working standard solutions of analytes and IS were obtained daily by diluting stock solutions in mobile phase. Mitra ^®^ VAMS™ 10 μl microsamplers were provided by Neoteryx (Torrance, CA, United States). HemaXis™ DB 10 devices were purchased from DBS System (Gland, Switzerland).

The HPLC system consisted of a Jasco (Tokyo, Japan) PU-2080 chromatographic pump and an Antec (DB Leiden, Netherlands) Decade amperometric detector equipped with a flow cell including a glass carbon working electrode and an Ag/AgCl reference electrode. The analytical cell was set at a potential of +800 mV, with a range of 20 nA. The detector was thermostatted at 27°C.

Chromatographic separation was performed on a Waters SunFire C18 reversed phase column (150 mm × 4.6 mm, 5 μm) coupled to a C18 reversed phase guard column (4 mm × 3 mm). The mobile phase was composed of ACN (15%, V/V), MeOH (20%, V/V), 26.6 mM phosphate buffer containing 3.2 mM KCl, and 0.4% of triethylamine (65%, V/V), and pH was adjusted to 2.50. Flow rate was set to 1 ml/min, while injection volume was 50 μl. Chromatographic data were analyzed by means of the Star Chromatographics 4.0 software (Varian, Palo Alto, CA, United States).

### Sample Collection and Pretreatment

Blank blood samples for method development and validation had already been collected for other research needs from healthy volunteers not undergoing any pharmacological treatment at the time of collection, and had been sampled both in fluid and in dried forms, as described below. Venous blood was obtained by phlebotomy in glass tubes containing EDTA as anticoagulant and was centrifuged at 4,000 rpm for 10 min at 4°C to obtain plasma. Spiked samples (fortified with the analytes and IS) were obtained by adding 5 μl of a mobile phase solution containing the analytes and IS to 95 μl of either whole blood or plasma.

**FIGURE 2:**
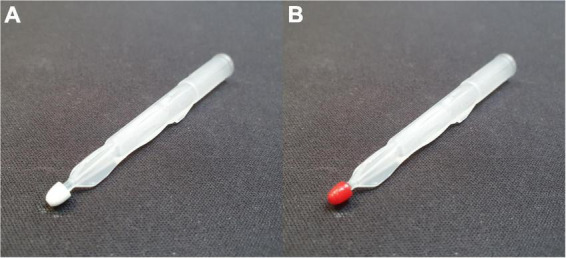
Ten-microliter VAMS devices, **(A)** blank and **(B)** used to sample a whole blood specimen by barely touching the fluid whole blood surface.

B-VAMS and p-VAMS samples were obtained from spiked whole blood and plasma by dipping the tip of the VAMS device in the sample until the tip was completely saturated; the tip was kept in contact with the matrix for an additional 2 s to ensure complete filling (Figure 2). Next, the VAMS samples were dried for 1 h prior to extraction. If samples were not analyzed within the same day, they were stored at RT in their specific clamshell container and zip-lock bags with desiccant until analysis.

With regard to mfDBS microsamples, a micropipette was used to fill HemaXis microfluidic channels with 10 μl of the spiked matrices described above. MfDBS samples were generated consecutively for four times by closing the protective case until matrix transfer from the microchannel to the DBS card was completed. Similarly to the VAMS samples, mfDBS samples were dried for 1 h prior to extraction or dried and stored at RT in zip-lock bags with desiccant until analysis.

The mode of IS addition to the samples was tested during method validation by comparing pre-collection fortification (as described above) and post-collection fortification. In the latter case, IS standard solution was added to dried VAMS and mfDBS samples right before extraction. The pre-sampling fortification approach with IS standard solution was aimed at providing comprehensive information about extraction performances, since analytes and IS are both integrated within matrix components and interacting with the support material, thus being co-extracted as parts of the sample. The post-sampling fortification approach with IS standard solution, on the other hand, was chosen in order to simulate real-life scenarios, where whole blood samples can be collected from a finger prick, barely touching the VAMS tip and the microfluidic channel to the blood drop on the fingertip, left to dry and shipped to the analysis laboratory. All the assays were carried out in triplicate.

Whole blood samples for method application were provided by in- and out-patients of different Italian psychiatric clinics and hospitals who were undergoing CLZ treatment and were collected for general needs related to the therapy. Institutional committee approval of the experiments was not required, since all the samples had already been collected for general needs related to the therapy and anonymized immediately after collection, and all the subjects provided informed consent prior to their participation in this study. After b-VAMS and mfDBS collection, whole blood from the same subjects was centrifuged, and p-VAMS samples were obtained as described above. After that, fluid plasma was transferred to polypropylene tubes and stored at −20°C until solid phase extraction (SPE) pretreatment, used as standard, reference plasma method for comparison with the results obtained from blood microsamples.

A VAMS tip or a whole mfDBS cut with a 9-mm puncher was transferred into a polypropylene tube together with 500 μl of MeOH (for both VAMS types) or 125 μl of a phosphate buffer/ACN/MeOH (52/12/36, V/V/V) mixture (for mfDBS). Then, the resulting mixture was subjected to ultrasound-assisted extraction (UAE) for 15 min. The mfDBS extract was directly injected into the HPLC-ED system, while the VAMS methanolic extract was dried under vacuum, re-dissolved with 125 μl of the mobile phase, and then injected into the HPLC-ED system.

To assess the reliability of the proposed dried microsampling approaches, the results provided by b-VAMS, p-VAMS, and mfDBS samples were compared to those from reference plasma analysis; plasma samples were subjected to SPE following an optimized procedure already validated as described in a previously published research article (40).

### Method Validation

The analytical method was validated according to European Medicines Agency (EMA) and United States Food and Drug Administration (FDA) guidelines (41, 42). For linearity, blood and plasma samples were fortified with CLZ, DCM, and NOX at seven different concentrations and IS at a constant concentration of 100 ng/ml before VAMS and mfDBS sampling. The pretreatment procedure previously described was applied to dried microsamples, which were then analyzed (*n* = 3). A calibration curve was set up using the least-square method by plotting the relative peak areas of the analyte/IS (pure numbers) against the corresponding analyte concentrations (expressed in ng/ml), and the lower limit of quantification (LLOQ) was set at the lowest calibrator level.

Extraction yield and precision were assessed on pretreated samples through HPLC-ED analysis by adding known amounts of CLZ, DCM, and NOX at LLOQ and three other different concentrations (corresponding to the low and intermediate levels of the calibration and to the upper limit of quantification, ULOQ) to fluid blood, collected by means of VAMS and HemaXis, and to plasma, collected by VAMS, pretreated and analyzed by HPLC-ED. Analyte peak areas were compared with those obtained by injecting standard solutions at the same theoretical concentrations to calculate extraction yield, expressed as percentage recovery. The assays were repeated six times on the same day and over six different days to obtain intra-day and inter-day precision, respectively, both were expressed as percentage relative standard deviation (RSD%).

Selectivity was evaluated by collecting, pretreating, and injecting blank microsamples from six different volunteers into the HPLC-ED system; the chromatogram of the blank samples and the LLOQ peak area of each analyte, at their respective retention time, were compared. Possible interfering CNS drugs were also injected at a concentration of 1 μg/ml. Selectivity was considered acceptable when any interfering peak at the same retention times as the analytes and IS was ≤ 20% of the LLOQ for each analyte and ≤ 5% of the mean IS area.

### Hematocrit Effect

In order to study a possible variation in analyte recovery with respect to HCT value, spiked whole blood samples were prepared with low (0.3), medium (0.5), and high (0.7) HCT levels and sampled to obtain both b-VAMS and mfDBS samples.

Extraction recoveries for the low- and high-HCT samples were evaluated in comparison to the medium-HCT samples. Recovery of the low- and high-HCT samples was considered independent from HCT if it was within ±15% of the recovery observed for medium-HCT.

### Stability

Stability assays were performed to ensure that analyte concentrations are not affected by storage conditions in dried microsamples stored at RT and in fluid matrices under freezing conditions (−20°C).

The stability of spiked b-VAMS, p-VAMS and mfDBS samples (*n* = 3) was assessed for 30 days at RT, with the samples stored in zip-lock bags with desiccant and protected from direct light. The measured analyte concentrations were compared to those of the same samples extracted and immediately analyzed after sampling and drying. The results were also compared to those obtained from fluid spiked plasma samples (*n* = 3) stored at −20°C for 30 days.

### Patient Samples

Following development and validation, the analytical methodology involving VAMS and mfDBS microsampling coupled to HPLC-ED analysis was applied to real samples from five patients undergoing therapy with CLZ. Patient whole blood samples were drawn into tubes containing EDTA as an anticoagulant and corresponding b-VAMS and mfDBS samples were obtained. Then, after whole blood centrifugation, the plasma was separated and used both to produce paired p-VAMS samples and to undergo SPE pretreatment and provide a benchmark for microsample performance.

Dried and fluid samples were pretreated and analyzed according to the procedures previously described. The quali-quantitative results obtained from dried microsample analysis were compared to those from the analysis of fluid plasma by linear regression using the least-square method to calculate the linearity correlation coefficient and slope of each curve and demonstrate the agreement between the data sets. Accuracy assays were performed by adding analyte standard mixtures at three concentrations (low, medium, and high) to p-VAMS, b-VAMS, and mfDBS patient sample replicates, whose analyte content had already been determined. Accuracy was expressed as percentage recovery of the spiked amount; the assays were repeated in triplicate.

## Results and Discussion

### Chromatographic Conditions

Chromatographic conditions were developed and optimized, starting from those previously used to analyze CLZ and its metabolites by HPLC-ED and modifying mobile phase composition and detector parameters in order to optimize the separation of chromatographic peaks and increase the sensitivity of the method (30). The resulting mobile phase was composed of ACN (15%, V/V), MeOH (20%, V/V), 26.6 mM phosphate buffer, and 3.2 mM KCl, and it contained 0.4% of triethylamine (65%, V/V); the pH was adjusted to 2.50. The injected volume was 50 μl. These conditions resulted in satisfactory performance, with sharp and symmetric peaks for CLZ, DMC, NOX, and IS. The separation of the analytes and IS was achieved in less than 13 min.

### Development of VAMS and mfDBS Sampling and Pretreatment

Several parameters were evaluated to optimize the use of the novel microsampling technologies proposed herein. As a first step, the accuracy and variability of sampling volume were considered. Previous studies carried out on different biological matrices demonstrated that there were no statistically relevant differences between volumes collected with 10-μl VAMS tips and those obtained with volumetric pipettes, confirming the good sampling accuracy of this approach for both blood and plasma (43, 44). Similarly, gravimetric tests were performed to define the volume of whole blood sampled with the HemaXis technology by weighing the DBS card before and after sampling and comparing the results with the weight of the same matrix sampled with a micropipette. The collected volumes are statistically indistinguishable from those pipetted (∼10 μl), thus proving the good sampling accuracy by means of microfluidic channel-based technology (9.7 ± 0.2 μl).

VAMS sampling and drying times were previously evaluated, and it was verified that contact times of ∼2 s were enough to completely fill 10-μl b-VAMS and p-VAMS tips. The results of drying time assays showed the achievement of constant weight (i.e., complete drying) in 1 h (25, 44). With regard to the microfluidic channel-based technology, on the other hand, total sampling time should be considered as the sum of two distinct steps: the time required to fill the microfluidic capillary, which ends when the channel outlet is full (about 2 s), and the time required to transfer blood to the paper card by gently pressing the two device panels, which ends when the channel is emptied and the spot is formed at the channel outlet (about 5 s). For shorter times, the capillary does not fill completely; therefore, the spot is not generated. If, on the other hand, the capillary does not empty completely, the spot is smaller, and the sampled volume is no longer accurate. In both cases, it is easy to notice that something has gone wrong: in the first case, in fact, no spots are formed, while in the second, residues of blood remain in the capillary. The time necessary for spot-drying was also studied: constant weight was reached in 1 h.

Then, pretreatment parameters influencing the extraction performance of VAMS and mfDBS were studied: extraction solvent, volume, time, and mode were optimized. Different amounts (125-1,000 μl) of pure solvents and solvent mixtures (methanol, acetonitrile, water, buffers, and their mixtures), extraction times (5-45 min), and extraction methods such as vortex-assisted extraction (VAE) and ultrasound-assisted extraction (UAE) were tested. The VAMS and mfDBS extraction procedures consisting respectively of 500 μl of MeOH and 125 μl of phosphate buffer/ACN/MeOH (52/12/36, V/V/V) by means of 15 min of UAE gave the best results (Supplementary Figure 1). In fact, for VAMS extraction, an increase in extraction yield was observed up to a volume of 500 μl; higher volumes did not bring any improvement in quantitative results but only increase in interferences. A similar behavior occurred for the time of UAE. The ratios of the mfDBS extraction mixture have been finely optimized together with the UAE time, which was gradually reduced from the initial time of 30 to 15 min to avoid extensive hemolysis and, thus, contamination of the extraction mixture. As can be seen, the use of these innovative VAMS and mfDBS microsampling procedures allowed to significantly reduce extraction times, solvent volumes, and biological sample manipulation in comparison to common extraction procedures from fluid samples.

### Method Validation

Standard solutions of analytes at seven different concentrations and IS at a constant concentration (100 ng/ml) were added to blank samples. The spiked blood and plasma were sampled with 10 μl b-VAMS, p-VAMS, and mfDBS, extracted, and analyzed in order to obtain calibration curves and calculate the LLOQ and limit of detection (LOD) for all the analytes and for the different matrices. All the linearity results are reported in Table 1.

**TABLE 1:** Linearity, LLOQ, and LOD results on VAMS and mfDBS spiked samples.

	p-VAMS				b-VAMS				mfDBS			
Analyte	Linearity range (ng/mL)	r^2^	LLOQ (ng/mL)	LOD (ng/mL)	Linearity range (ng/mL)	r^2^	LLOQ (ng/mL)	LOD (ng/mL)	Linearity range (ng/mL)	r^2^	LLOQ (ng/mL)	LOD (ng/mL)
CLZ	3–2,000	0.9993	3.0	1.0	3–2,000	0.9992	3.0	1.0	3–2,000	0.9991	3.0	1.0
DMC	3–2,000	0.9995	3.0	1.0	3–2,000	0.9991	3.0	1.0	3–2,000	0.9987	3.0	1.0
NOX	3–2,000	0.9989	3.0	1.0	3–2,000	0.9988	3.0	1.0	3–2,000	0.9992	3.0	1.0

Blank blood and plasma were fortified with four different concentrations of the analytes (LLOQ, 50, 500 ng/ml, and ULOQ, and IS at the constant concentration of 100 ng/ml) to evaluate extraction yield and precision. The results were satisfactory, as b-VAMS and p-VAMS showed results higher than 83.4 and 85.1%, respectively, while mfDBS extraction yield was always higher than 74.0% for all the analytes. Precision was also good, with RSD values lower than 5.4% for b-VAMS, 5.1% for p-VAMS, and 5.6% for mfDBS. The complete results of these assays are reported in Table 2.

**TABLE 2:** Extraction yield and precision results of spiked b-VAMS, p-VAMS, and mfDBS samples.

		p-VAMS			b-VAMS			mfDBS		
Analyte	Concentration level	Extraction yield (%)	Precision (RSD%)		Extraction yield (%)	Precision (RSD%)		Extraction yield (%)	Precision (RSD%)	
			Intraday	Interday		Intraday	Interday		Intraday	Interday
CLZ	LLOQ	87.1	4.3	5.0	85.0	4.5	5.3	90.2	4.7	5.2
	Low	89.0	3.9	4.1	88.5	4.0	4.6	99.7	4.3	4.6
	Intermediate	93.5	3.8	4.5	89.2	3.8	4.4	100.7	4.0	4.5
	ULOQ	90.3	3.5	4.3	90.6	3.8	4.0	101.5	3.6	4.2
DMC	LLOQ	85.2	4.4	4.7	83.5	4.5	4.8	84.3	4.6	5.0
	Low	89.2	3.9	4.0	88.0	4.2	4.5	85.3	4.4	4.7
	Intermediate	99.0	3.5	3.8	96.2	3.9	4.1	86.3	4.1	4.4
	ULOQ	98.2	3.2	3.5	97.3	3.7	3.8	87.4	3.8	4.0
NOX	LLOQ	89.0	4.5	4.9	87.4	5.0	5.3	74.1	5.0	5.5
	Low	94.8	4.0	4.5	90.2	4.5	4.8	75.1	4.6	5.1
	Intermediate	97.3	3.8	4.2	93.4	3.9	4.5	75.7	4.3	4.8
	ULOQ	97.2	3.8	4.0	93.1	4.0	4.2	76.8	4.2	4.6

In order to evaluate method selectivity, standard solutions of several CNS drugs potentially co-administered to psychiatric patients were injected into the HPLC system, such as valproate, duloxetine, olanzapine, haloperidol, trazodone, aripiprazole, quetiapine, biperidene, paroxetine, clotiapine, venlafaxine, vortioxetine, promazine, and gabapentin. None of these drugs were detected at retention times that could lead to interference with analyte or IS detection and quantitation. Moreover, the analysis of blank VAMS and mfDBS from 6 different healthy volunteers not subjected to pharmacological treatment showed no evidence of signals from endogenous compounds at the retention times of CLZ, DMC, NOX, or the IS.

Extraction yield assays, carried out to compare the effectiveness of IS addition either before or after microsampling, provided strictly overlapping results (r^2^ values ≥ 0.999, *n* = 3), thus demonstrating the promising applicative suitability of both approaches.

### Hematocrit Assays

**FIGURE 3:**
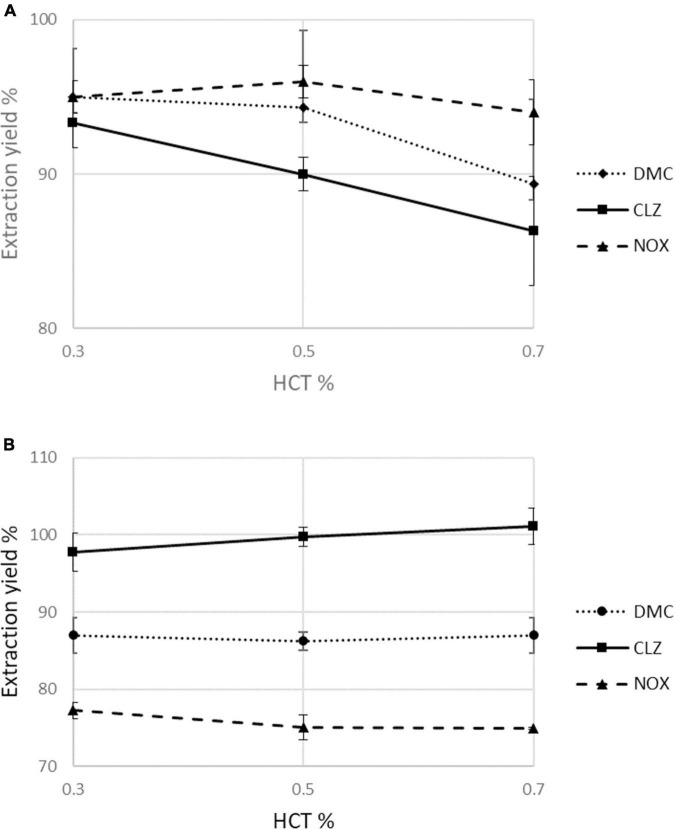
Assessment of the effect of hematocrit values (0.3, 0.5, and 0.7) on mean percentage extraction yields in **(A)** b-VAMS and **(B)** mfDBS.

To evaluate in depth the possible influence of HCT in terms of extraction yield, three representative HCT values (0.3, 0.5, and 0.7) for each analyte were considered. For DMC, CLZ, and NOX extracted from b-VAMS, the extraction yields increase slightly at lower HCT levels, while in the mfDBS samples, the small variations in extraction yields are dependent on the nature of the analytes. Overall, the extraction yields resulted to be HCT-independent (i.e., always within ± 15% of the recovery from blood with 0.5 HCT), as reported in Figure 3.

### Stability

The stability of the analytes in fluid plasma samples under controlled conditions (−20°C) was compared with that in the dried matrices, stored at RT, in the presence of drying agents and protected from direct light (n = 3). After 30 days, the VAMS and mfDBS samples showed higher stability (mean analyte loss <4.5, <4.6, and <4.9% for p-VAMS, b-VAMS, and mfDBS, respectively) than the plasma samples (mean analyte loss <6.3%), despite the VAMS and mfDBS samples being stored at RT. Therefore, a significant improvement in analyte stability was obtained with the micromatrices, as the drying process reduces analyte degradation by stopping most enzymatic and chemical reactions.

### Analysis of Blood and Plasma From Psychiatric Patients

**FIGURE 4:**
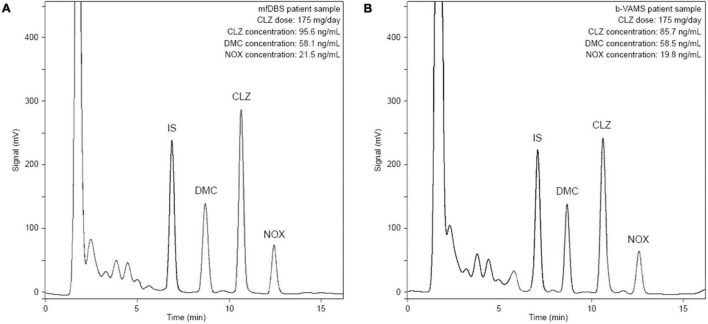
Chromatograms obtained from the analysis of **(A)** mfDBS and **(B)** b-VAMS samples from a psychiatric patient treated with 175 mg/day of CLZ.

The analytical methodology was applied for the analysis of real samples from 5 patients undergoing CLZ therapy. Whole blood was collected in glass tubes containing EDTA as an anticoagulant 12 h after the last administration of CLZ. In addition to mfDBS, b-VAMS and p-VAMS samples were obtained before and after centrifugation, respectively, and stored at RT until pretreatment and analysis. Representative chromatograms from the analysis of an mfDBS and a b-VAMS sample obtained from the same psychiatric patient treated with 175 mg/day of CLZ are reported in Figures 4A,B, respectively.

Quali-quantitative results obtained from patient VAMS and mfDBS samples were compared to those from fluid plasma samples, subjected to the SPE pretreatment procedure, to evaluate the performance of the dried microsampling approach. As reported in Table 3, all results from the micromatrices are in very good agreement with those from fluid plasma, such as for p-VAMS (r^2^ value ≥ 0.9991) and after conversion factor application in the case of mfDBS (r^2^ value ≥ 0.9983) and b-VAMS (r^2^ value ≥ 0.9995) to account for the presence of erythrocytes in these micromatrices (45). A graphical representation of these results is shown in Supplementary Figure 2. Finally, accuracy was evaluated, and satisfactory results were obtained for all the matrices, in the 91-108 and 93-105% ranges for b-VAMS and p-VAMS, respectively, and in the 89-109% range for mfDBS.

**TABLE 3:** Quantitative comparisons among wet and dried samples obtained from patients undergoing CLZ therapy.

Drug dose (mg/day)	Analyte	Plasma concentration found (ng/mL)			
		Fluid plasma	p-VAMS	b-VAMS	mfDBS
175	CLZ	102.7	100.8	85.7	95.6
	DMC	53.5	54.6	58.5	58.1
	NOX	14.3	18.7	19.8	21.5
200	CLZ	239.1	230.9	232.0	234.7
	DMC	116.8	108.7	101.4	114.9
	NOX	28.1	31.7	36.8	26.4
300	CLZ	283.7	268.1	279.6	275.7
	DMC	232.5	220.3	240.2	218.3
	NOX	49.5	40.5	45.6	42.0
300	CLZ	280.0	270.0	295.8	243.0
	DMC	109.1	125.6	105.8	104.5
	NOX	44.4	42.3	49.2	32.5
500	CLZ	1666.7	1620.5	1640.2	1634.5
	DMC	607.3	548.9	602.1	536.4
	NOX	156.7	153.7	162.9	151.7

## Conclusion

Original and innovative methodologies based on b-VAMS, p-VAMS, and mfDBS microsampling approaches coupled to HPLC-ED (amperometric detector) have been developed and validated for measurement of CLZ and its main metabolite levels in micromatrices. The significant advantages of these innovative technologies in terms of minimal sampling invasiveness, reduction of matrix volume, storage and transport temperature, and feasibility of pretreatment procedure, allowed the optimization of a straightforward and high-throughput strategy to carry out the TDM of psychiatric patients undergoing CLZ treatment. Both microsampling technologies proved to be effective, with each one showing different practical strengths. This allowed to derive some useful practical information to get the most out of each of the two microsampling technologies.

For example, it is easy to visually check when the tip of VAMS is fully saturated, especially with whole blood, while during the filling of the HemaXis channel, attention should be paid to the formation of bubbles if the filling is interrupted and more contacts are made with the fingertip blood drop, but this risk does not exist with VAMS. The two HemaXis panels have to be closed after each sampling because of the capillaries not being coated with an anticoagulant to allow spot generation. This may determine the need to perform more finger pricks to obtain 4 consecutive replicates. On the other hand, the developed pretreatment procedure for mfDBS is extremely fast and does not include solvent evaporation and reconstitution. The collection of p-VAMS is more laborious, and sampling cannot be performed through at home- and self-sampling procedures, as whole blood centrifugation is necessary. Both approaches involve a transport and storage casing that can prevent any sample contamination. Overall, sampling speed is much increased with finger pricking instead of venipuncture, also providing the chance to exploit this procedure for self-sampling and at-home sampling, with obvious time and cost advantages. Considering that the microsamples do not require controlled temperatures for transport and storage, and smaller storage space with respect to the corresponding fluid macrosamples, the proposed approach could lead to major advantages in cost-effectiveness and logistics for routine clinical applications.

Satisfactory validation results were obtained for all the microsamples: extraction yield > 83.4%, RSD < 5.4%, stability with analyte loss < 4.6% after 30 days for b-VAMS, extraction yield > 74%, RSD < 5.6%, and stability with analyte loss < 4.9% after 30 days for mfDBS, but the best extraction yields were shown by the p-VAMS approach with recovery values > 85.1%, RSD < 5.1%, and stability with analyte loss < 4.5% after 30 days. An in-depth HCT effect study was performed to evaluate its possible influence on recovery; both the VAMS and mfDBS strategies met the acceptance criteria (within ± 15% of the recovery from blood with mean HCT value).

The amperometric detector combines better sensitivity and selectivity if compared with other detectors such as UV or DAD while granting much lower acquisition costs, and avoiding the need for specific personnel training with respect to LC-MS/MS methodologies, currently considered state-of the-art for drug analysis. The excellent performances observed with this setup are further proof of microsample approach soundness and of the feasibility of microsampling for CLZ monitoring. The optimized workflow was applied for the TDM of psychiatric patients undergoing CLZ therapies, with the determination of the parent drug and its two main metabolites in dried micro-matrices conducted by b-VAMS, p-VAMS, and mfDBS.

In order to assess the reliability of the miniaturized methodology, classic fluid plasma analysis was also performed, showing satisfactory overlapping of results between the “wet” and dried matrices (r^2^ values ≥ 0.9983), thus the results of this study confirm that the developed protocols are accurate and reliable. In conclusion, blood and plasma microsampling represents a mature platform for possible implementation as a routine procedure, particularly in the perspective of home- and self-sampling for frequent TDM of patients taking CLZ and, from a future perspective, other antipsychotic drugs.

## Data Availability Statement

The original contributions presented in the study are included in the article/Supplementary Material, further inquiries can be directed to the corresponding author.

## Ethics Statement

Ethical review and approval was not required for the study on human participants in accordance with the local legislation and institutional requirements. The patients/participants provided their written informed consent to participate in this study.

## Author Contributions

CM carried out the analysis and method validation, and wrote the original draft. RM provided resources, conducted data curation, and reviewed and edited the final version of the manuscript. RS conducted data and review and editing of the final version of the manuscript. TV helped with conceptualization and conducted data curation. AA provided resources and reviewed and edited the final version of the manuscript. AC provided resources and acquired funding. AS provided clinical expertise and reviewed and edited the final version of the manuscript. MP conducted project conceptualization and supervision, data curation, writing of the original draft, and review and editing of the final version of the manuscript. LM conducted project conceptualization, supervision and administration, acquisition of funding and resources, and review and editing of the final version of the manuscript. All authors have read and approved the final version of the manuscript.

## Conflict of Interest

The authors declare that the research was conducted in the absence of any commercial or financial relationships that could be construed as a potential conflict of interest.

## Publisher’s Note

All claims expressed in this article are solely those of the authors and do not necessarily represent those of their affiliated organizations, or those of the publisher, the editors and the reviewers. Any product that may be evaluated in this article, or claim that may be made by its manufacturer, is not guaranteed or endorsed by the publisher.
