# Supplementary material for: Dried Volumetric Microsampling Approaches for the Therapeutic Drug Monitoring of Psychiatric Patients Undergoing Clozapine Treatment
Source: Front Psychiatry. 2022 Jun 1;13:794609. doi: 10.3389/fpsyt.2022.794609 (PMC9198272; doi:10.3389/fpsyt.2022.794609)
Supplement: Supplementary file 1 [file Data_Sheet_1.pdf]

# *Supplementary Material*

## Supplementary Figures

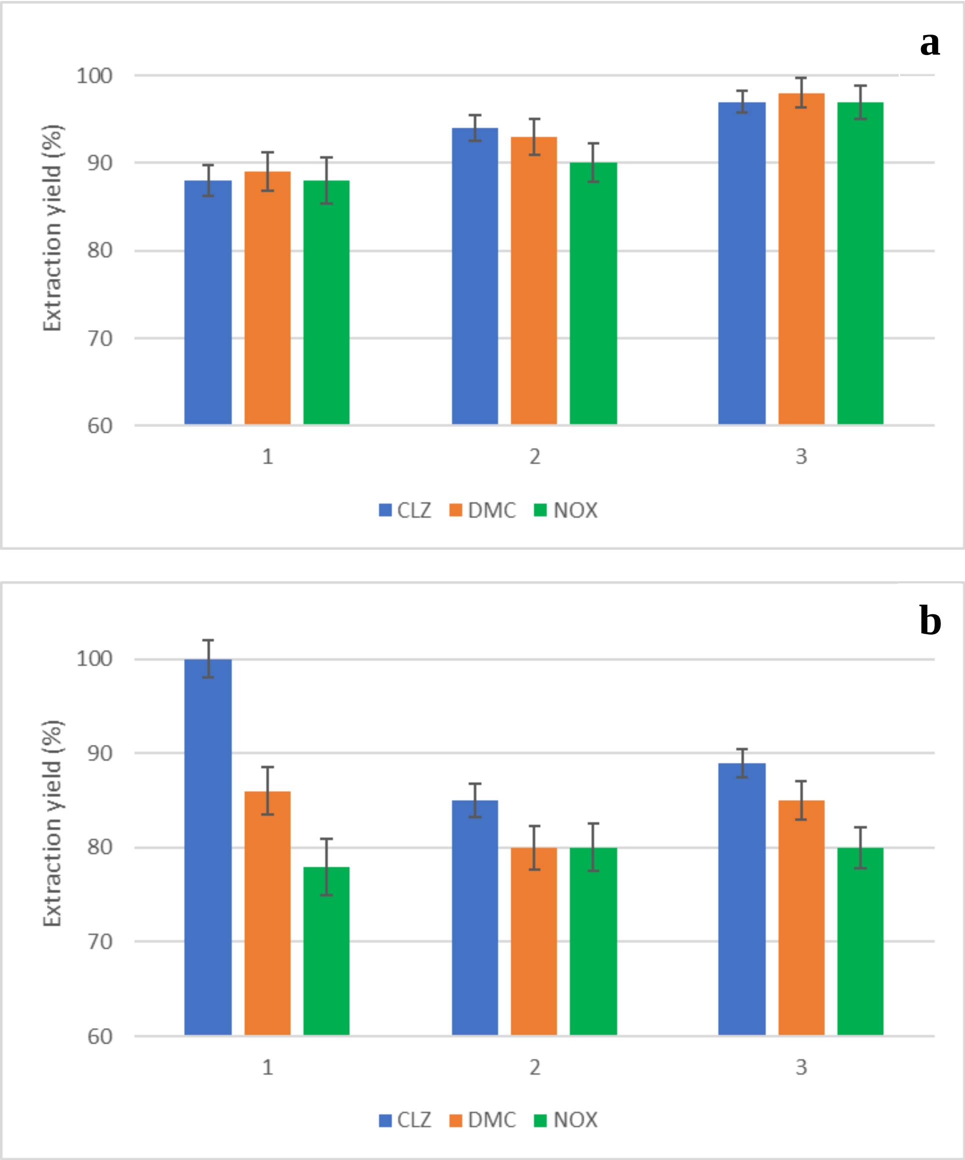


**Supplementary Figure 1.** Extraction solvents and solvent mixtures tested for (a) VAMS and (b) mfDBS extraction: 1: Phosphate buffer/ACN/MeOH, 52/12/36 (V/V/V); 2: ACN/MeOH 1:1 (V/V); 3: 100% MeOH.

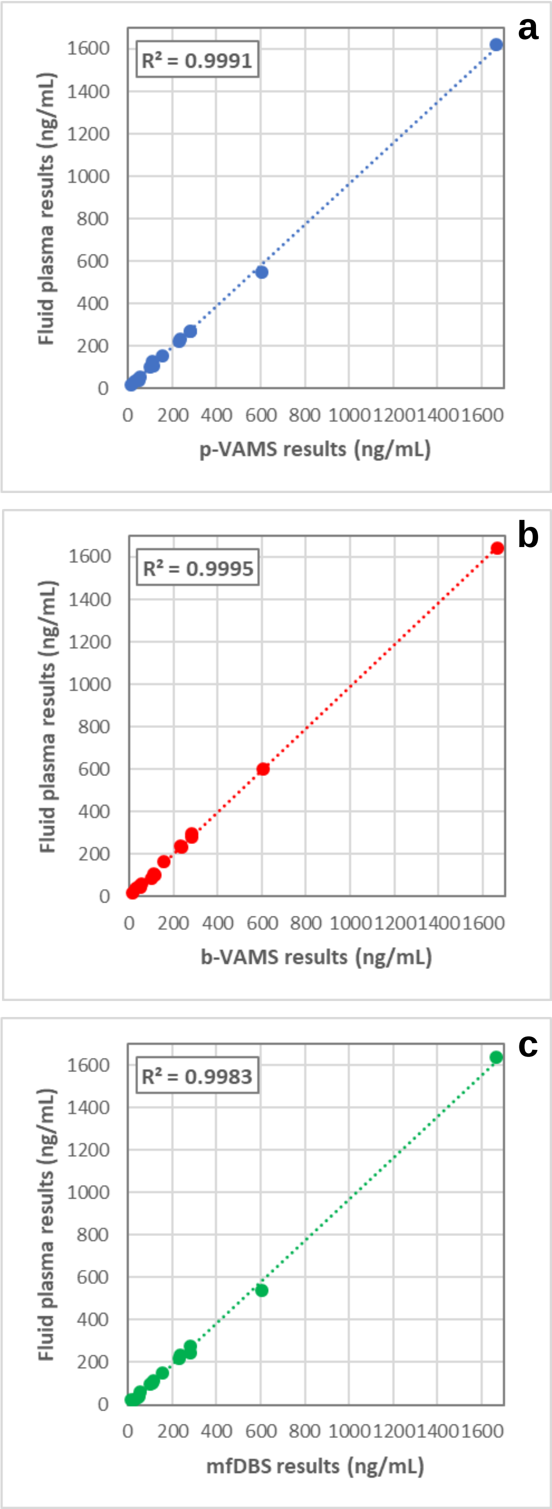


**Supplementary Figure 2.** Linear regression graphs for the comparisons of plasma concentrations obtained from fluid plasma with those obtained from (a) p-VAMS, (b) b-VAMS and (c) mfDBS.
